# CDH6 as a prognostic indicator and marker for chemotherapy in gliomas

**DOI:** 10.3389/fgene.2022.949552

**Published:** 2022-07-22

**Authors:** Ming Meng, Hongshu Zhou, Ye He, Lu Chen, Wanpeng Wang, Liting Yang, Zeyu Wang, Liyang Zhang, Sha Wang

**Affiliations:** ^1^ Department of Neurosurgery, Xiangya Hospital, Central South University, Changsha, China; ^2^ National Clinical Research Center for Geriatric Disorders, Xiangya Hospital, Central South University, Changsha, China; ^3^ Eye Center of Xiangya Hospital, Central South University, Changsha, China; ^4^ Hunan Key Laboratory of Ophthalmology, Changsha, China; ^5^ Clinical Diagnosis and Therapy Center for Glioma, Xiangya Hospital, Central South University, Changsha, China

**Keywords:** CDH6, marker, chemotherapy, glioma, prognosis

## Abstract

Glioma is the most malignant cancer of the central nervous system. There are various therapies for treating gliomas, but their outcomes are not satisfactory. Therefore, new targets for glioma treatment are needed. This study examined the cadherin-6 (CDH6) expression in gliomas using The Cancer Genome Atlas and Chinese Glioma Genome Atlas datasets. CDH6 expression positively correlated with the World Health Organization (WHO) tumor grade and negatively correlated with patient prognosis. A significant decrease in CDH6 promoter methylation was identified with an increase in the WHO grade severity. Gene Ontology and Kyoto Encyclopedia of Genes and Genomes enrichment analyses suggested that CDH6 might be involved in cell–cell interactions and immune processes in the glioma microenvironment. Weighted gene co-expression network analysis revealed a correlation between CDH6 and cell adhesion molecules, focal adhesions, phosphatidylinositol 3-kinase-protein kinase B signaling pathways, nuclear division, chromosome segregation, mitotic nuclear division, and immune-related pathways. CDH6 strongly correlated with immunosuppressive cells, including regulatory T cells, monocytes, macrophages, tumor-associated macrophages, and myeloid-derived suppressor cells. It also showed correlations with immune-active cells such as B cells, CD8^+^ T cells, and dendritic cells. Single-cell analysis showed that CDH6 was expressed mainly in astrocyte (AC)-like malignant cells. Differentially expressed genes of AC-like malignant cells were found to be associated with stress response, membranous processes, viral infections, and several types of cancers. Potential drugs associated with high CDH6 expression were also predicted, including AMG-22, rutin, CCT128930, deforolimus, bis(maltolato)oxovanadium, anagrelide, vemurafenib, CHIR-98014, and AZD5582. Thus, this study showed that CDH6 correlates with glioma immune infiltration, it is expressed mainly in AC-like malignant cells, and it may act as a new target for glioma therapy.

## Introduction

Glioma is one of the most common and malignant cancers of the central nervous system in humans. According to the World Health Organization (WHO) criteria, gliomas are classified into grades Ⅰ–Ⅳ. Glioblastoma multiforme (GBM) is the most malignant type of glioma and has poor prognosis. Patients with GBM have a 5-years survival rate of less than 10% (Stupp et al., 2009; Weller et al., 2017). Currently, therapies for GBM include surgical resection, radiation, chemotherapy, immunotherapy, and tumor treating fields, but they do not prolong patient survival significantly (Janjua et al., 2021). The development of immunotherapy has improved the treatment outcome in several cancers in humans (Kim et al., 2020; Wang et al., 2021). Immune checkpoint inhibitors, such as anti-programmed death-1 (PD-1), and anti-cytotoxic T lymphocyte antigen 4 (CTLA4) therapies enhance the activity of T cells and inhibit immunosuppression in the tumor microenvironment (Ribas and Wolchok, 2018; Liu et al., 2020).

The presence of certain biomarkers, such as mutated isocitrate dehydrogenase (IDH), O6-methylguanine DNA methyltransferase (MGMT) promoter hypermethylation, epidermal growth factor receptor amplification, and p53 mutations in GBM offer prognostic and diagnostic potential. Therefore, it is promising to identify additional potential molecular targets for the diagnosis and treatment of GBM to improve patient prognosis (Zhang et al., 2020).

The cadherin (CDH) family comprises calcium-dependent transmembrane proteins responsible for cell–cell adhesion during embryogenesis, tissue morphogenesis, differentiation, and maintenance of normal tissue architecture (Yulis et al., 2018; Kaszak et al., 2020). Recent studies have confirmed that CDH-mediated signaling plays a key role in development, proliferation, apoptosis, and disease pathobiology (Arulanandam et al., 2009; Harris and Tepass, 2010; Hawkins et al., 2012; Bektas et al., 2013). Over the past few decades, the role of CDHs has been evaluated in many malignancies, such as melanoma, hepatocellular carcinoma, breast cancer, and gastric cancer (Kaszak et al., 2020). In a previous study, despite isolation of glioma patient-derived tumor cells (GPDCs), characteristic genomic features and potential therapeutic markers were reported (Zhang et al., 2021). CDH6 is a type II cadherin containing five extracellular domains and one cytoplasmic domain that facilitate its interaction with catenin molecules (Casal and Bartolomé, 2019). *ß*-Catenin binds directly to the cytoplasmic tail of CDHs and to α-catenin to regulate the actin cytoskeleton. CDH6 is believed to play a role in the interaction between cell adhesion and *ß*-catenin. CDH6 has been implicated in various processes, including epithelial-mesenchymal transition (EMT), autophagy (Gugnoni et al., 2017), and metastasis (Bartolomé et al., 2021). We also found that the transcriptional level of CDH6 significantly increased in high-grade GPDCs. This indicates that CDH6 may play an essential role in glioma tumor progression and interactions with the microenvironment components. In this study, high CDH6 expression was predictive of poor patient prognosis. Weighted gene co-expression network analysis (WGCNA) revealed a correlation between CDH6 and cell adhesion molecules, focal adhesions, phosphatidylinositol 3-kinase-protein kinase B (PI3K-Akt) signaling pathways, nuclear division, chromosome segregation, mitotic nuclear division, and immune-related pathways. Single-cell analysis revealed that CDH6 was expressed mainly in astrocyte (AC)-like malignant cells. Additionally, potential drugs associated with high CDH6 expression were also predicted.

## Results

### Correlation of CDH6 expression with clinicopathological characteristics and prognosis in patients with glioma

**FIGURE 1:**
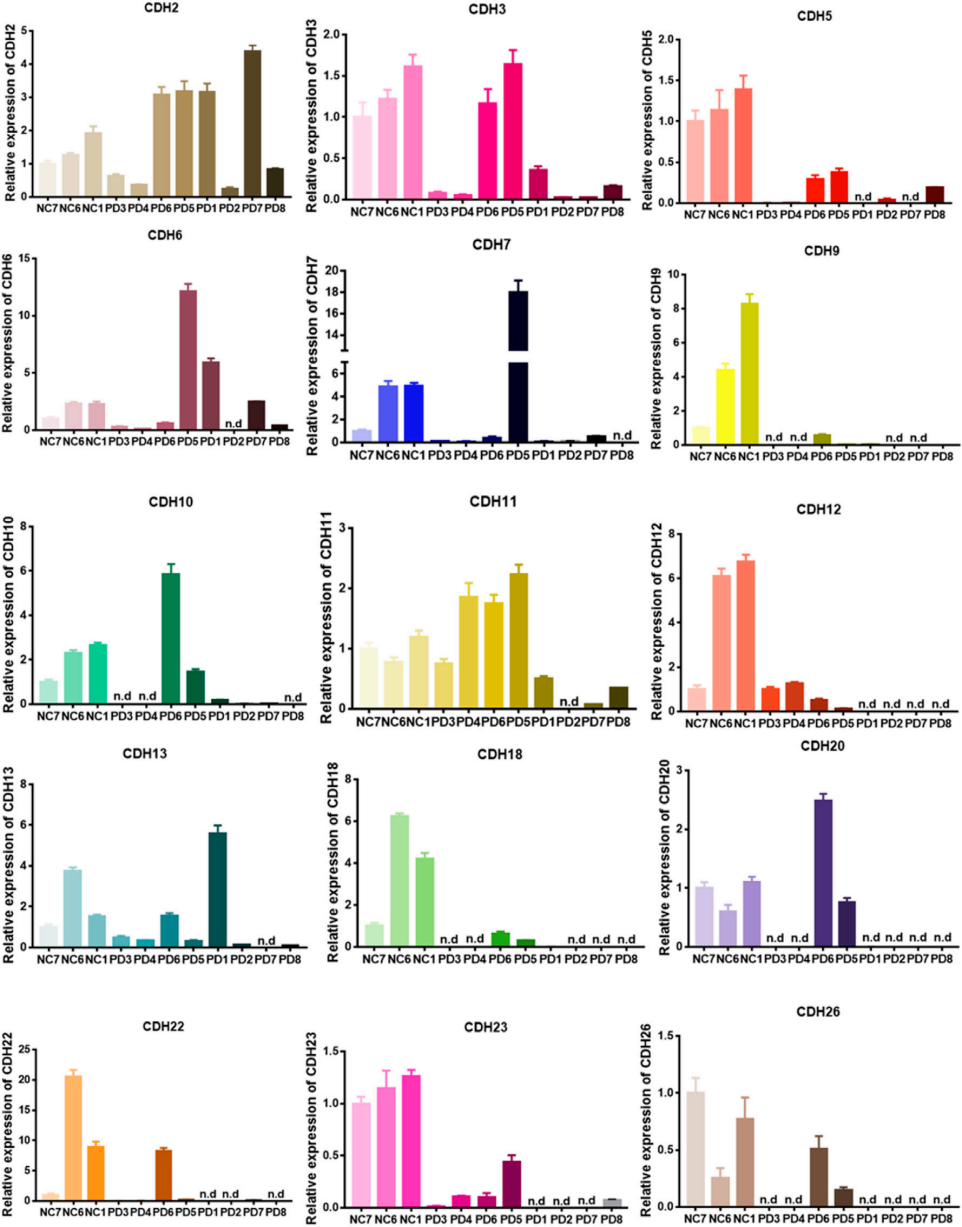
Real-time qPCR of patient derived glioma tissues.

**FIGURE 2:**
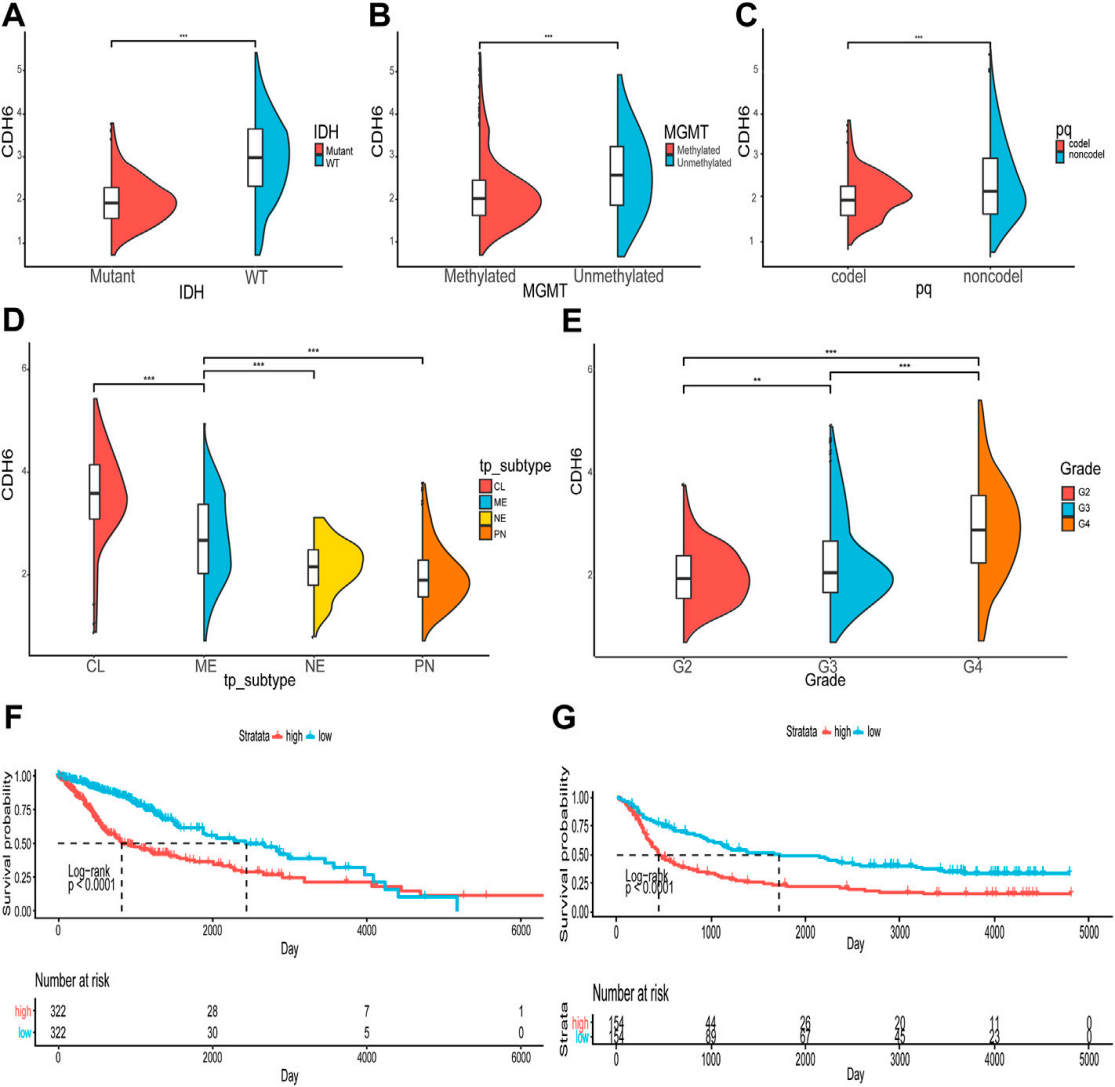
Correlation of CDH6 expression with clinicopathological characteristics. **(A–E)** CDH6 expression in different clinicopathological subgroups. **(F, G)** Survival analyses of high and low CDH6 expression subgroups in TCGA and CGGA datasets. *p < 0.05, **p < 0.01, ***p < 0.001.

We first examined the expression of the CDH family proteins using glioma tissues collected from patients. Gliomas of different grades exhibited differential expression of the CDH family proteins. CDH6 levels in gliomas differed significantly in our collected samples (Figure 1). Then we examined CDH6 expression in subgroups of various clinicopathological characteristics, including IDH mutation status, MGMT promoter methylation status, 1p19q codeletion status, and WHO grade. CDH6 expression was significantly higher in IDH wild-type, MGMT unmethylated, 1p19q non-co-deleted, and WHO Grade III and IV subgroups (Figures 2A–C, E). We also evaluated CDH6 expression in the GBM subgroups defined by Verhaak et al. (Verhaak et al., 2010). The classical subtype of GBM harbored the highest CDH6 expression (Figure 2D). IDH mutation is a principal marker of low-grade glioma (LGG), while MGMT promoter methylation is a predictor of temozolomide drug response. Data from CGGA325 and GSE108474 exhibited similar results (Supplementary Figures S1, 2). These results indicate a latent role of CDH6 in glioma diagnosis and therapeutics.

The correlation between CDH6 expression and patient prognosis was explored (Figures 2F,G). Patients were divided into high and low CDH6 expression subgroups based on the median CDH6 expression. In The Cancer Genome Atlas (TCGA) LGG–GBM and Chinese Glioma Genome Atlas (CGGA) 325 datasets, patients with low CDH6 expression showed a significantly longer overall survival than those with high CDH6 expression (*p* < 0.0001).

### Regulation of promoter methylation in CDH6 mRNA expression

**FIGURE 3:**
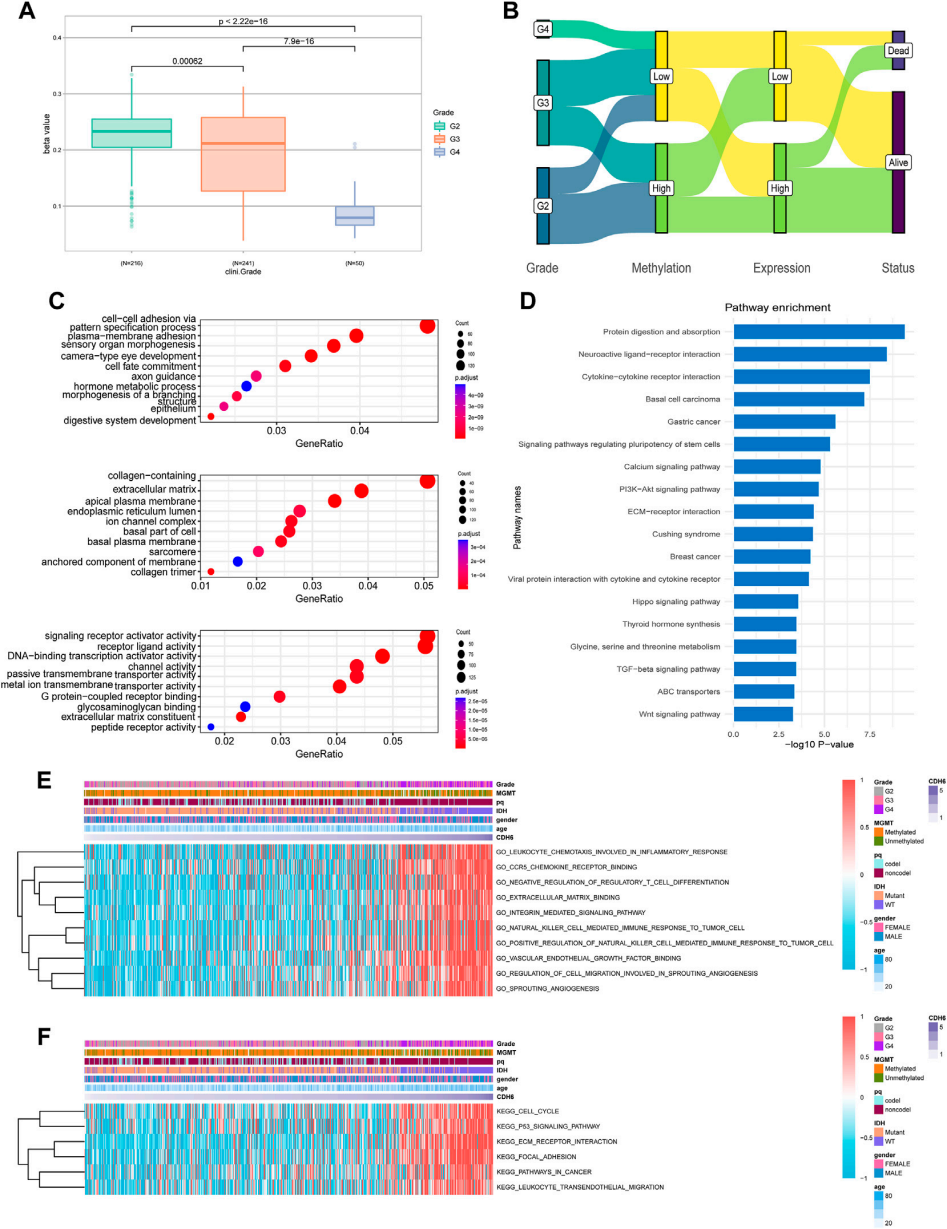
CDH6 methylation and enrichment analyses. **(A)** CDH6 promoter methylation in different grades. **(B)** Sankey plot of CDH6 promoter methylation, CDH6 expression and patient prognosis. **(C,D)** GO and KEGG analyses of CDH6 high expression subgroup. **(E,F)** GSVA analyses of CDH6 expression subgroups.

Promoter methylation is a common regulatory mechanism in mRNA expression. CDH6 promoter methylation was investigated using TCGA LGG–GBM dataset. A significant decrease in CDH6 promoter methylation was associated with an increase in the WHO grade severity (*p* = 0.00062, 7.9e-16, and <2.22e-16, respectively) (Figure 3A). The status of CDH6 promoter methylation, expression, and survival was investigated for the different WHO tumor grades (Figure 3B). Hypomethylation of the CDH6 promoter potentially leads to high expression of CDH6 and poor prognosis in glioma patients.

### CDH6 pathway enrichment analyses

We performed Gene Ontology (GO) and Kyoto Encyclopedia of Genes and Genomes (KEGG) analyses to identify pathways associated with CDH6 expression. GO analysis indicated multiple terms, including “cell–cell adhesion via plasma membrane adhesion,” “plasma membrane adhesion molecules,” and “collagen-containing extracellular matrix” (Figure 3C). KEGG analysis identified enriched pathways including “protein digestion and absorption,” “neuroactive ligand-receptor interaction,” “cytokine-cytokine receptor interaction,” and “PI3k-Akt signaling pathway” (Figure 3D). We also performed gene set variation analysis (GSVA) to uncover enriched pathways associated with CDH6 expression and acquired pathways, including “leukocyte chemotaxis involved in inflammatory response,” “C-C chemokine receptor 5 (CCR5) binding,” and “extracellular matrix binding” (Figures 3E,F and Supplementary Figure S3). These results suggest that CDH6 might be involved in cell–cell interactions and immune processes in the glioma microenvironment.

### Identification of CDH6 correlated genes in glioma

**FIGURE 4:**
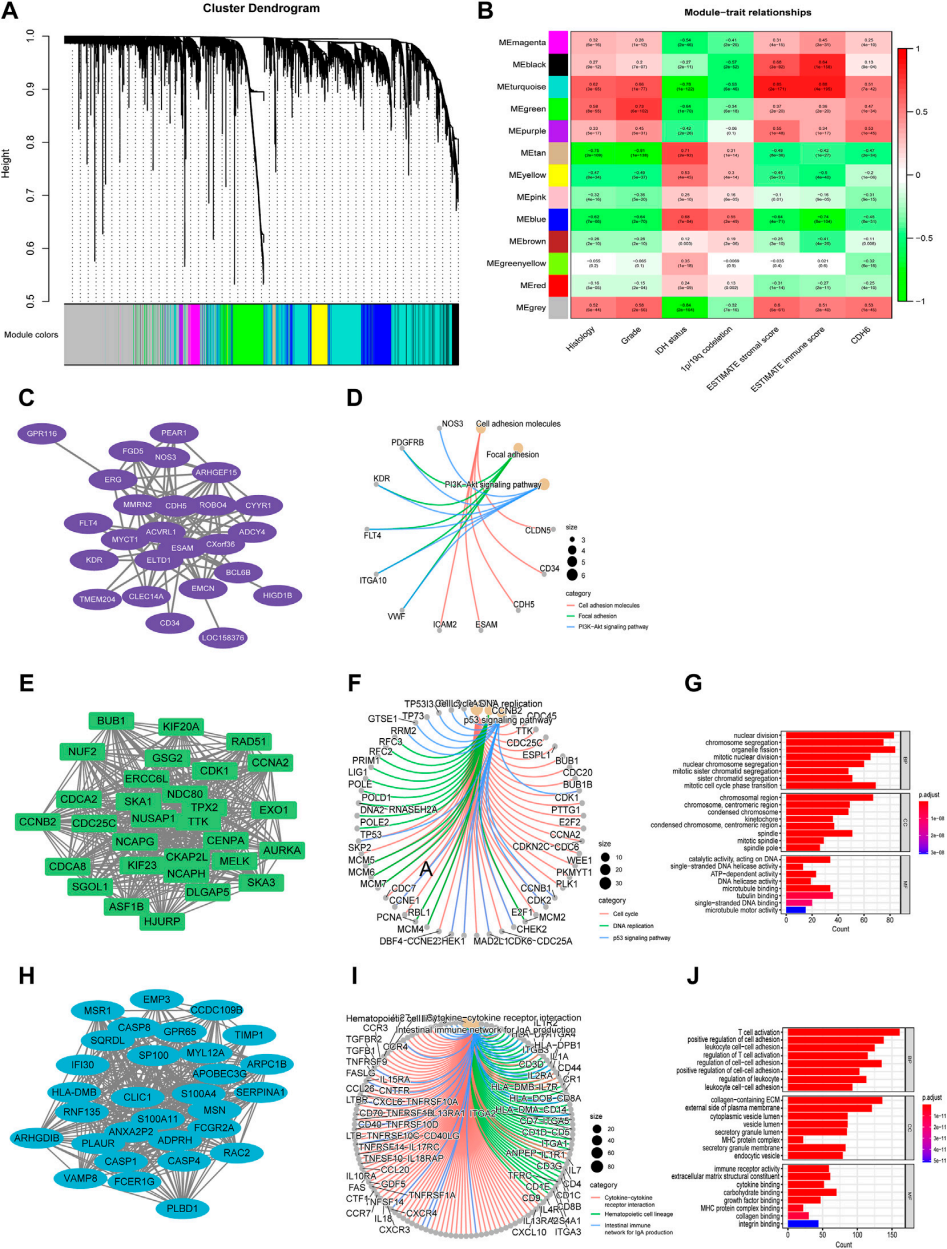
Identification of gene modules associated with CDH6 expression with WGCNA. **(A)** Cluster dendrogram of genes and clinicopathological variables. **(B)** Module-trait associations with rows corresponding to module gene sets and columns to traits. **(C,D)** PPI network and circus plot of the KEGG pathway analysis for the purple module. **(E–G)** PPI network, circus plot and barplot of the GO and KEGG analysis for the green module. **(H–J)** PPI network, circus plot and barplot of the GO and KEGG analysis for the turquoise module.

To identify gene modules associated with CDH6 expression, the top 10,000 median absolute deviation (MAD) genes were used in WGCNA. The genes were clustered into 13 modules (Figures 4A,B). We selected turquoise, green, and purple modules that were highly correlated with CDH6 expression for the downstream GO and KEGG pathway enrichment analyses (Figures 4C–J). The purple module genes were enriched in cell adhesion molecules, focal adhesions, and PI3K-Akt signaling pathways. The green module genes were enriched in pathways associated with cell division and DNA replication, including nuclear division, chromosome segregation, and mitotic nuclear division. The turquoise module genes were enriched in immune-related pathways, including T cell activation, leukocyte cell–cell adhesion, regulation of cell–cell adhesion, and collagen-containing extracellular matrix.

### Association of CDH6 with immune cell infiltration and cytokines

**FIGURE 5:**
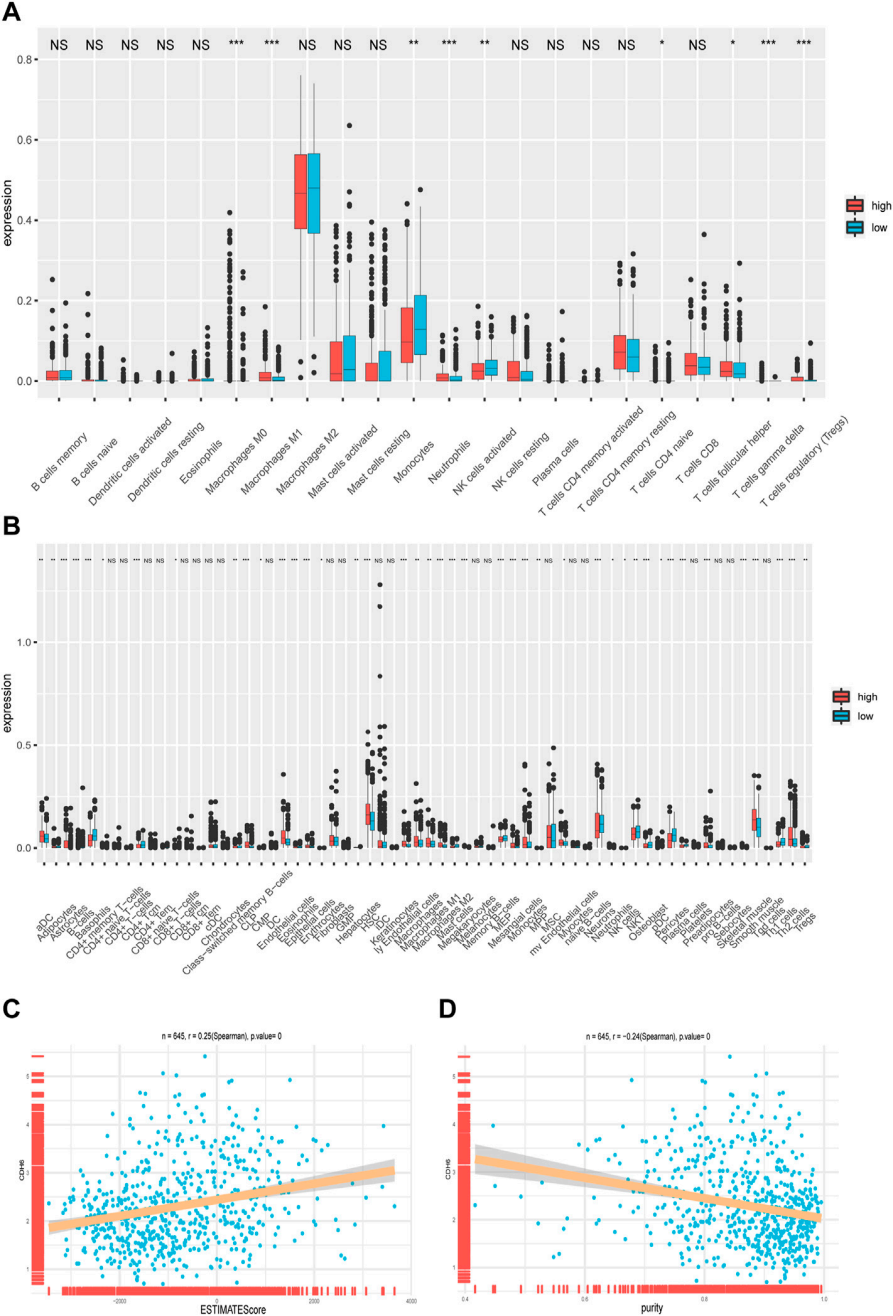
Correlation of CDH6 expression with immune cell infiltration. **(A)** Infiltration of 22 immune cells in CDH6 high and low expression subgroups. **(B)** Infiltration of 28 immune cells in CDH6 high and low expression subgroups. **(C,D)** Correlation of CDH6 expression with ESTIMATE score and tumor purity. *p < 0.05, **p < 0.01, ***p < 0.001, NS no significance.

The CIBERSORTx algorithm is an analytical tool that estimates immune cell abundance using gene expression data. We examined the infiltration of 22 types of immune cells in the subgroups categorized based on the median value of CDH6 expression (Figure 5A). M2 macrophages exhibited the highest infiltration rate among all the immune cells but showed no significant difference between the low and high CDH6 expression subgroups. Monocytes showed an overall high infiltration and a significantly higher infiltration in the low CDH6 expression subgroup than in the high CDH6 expression subgroup. Other immune cells showing significant differences between the CDH6 expression subgroups included M0 macrophages, M1 macrophages, neutrophils, activated natural killer (NK) cells, naïve CD4 T cells, follicular helper T (Tfh) cells, gamma delta T (T*γδ*) cells, and regulatory T cells (Tregs). The underlying mechanism of the immune infiltration difference between the high and low CDH6 expression subgroups warrants further investigation.

We also examined the infiltration of 28 subpopulations of tumor-infiltrating lymphocytes (TILs) from The Cancer Imaging Archive database (Figure 5B), which yielded similar results. TILs are divided into adaptive and innate immunity cohorts, according to the immunological processes with which they are associated. The adaptive immunity cohort includes activated T cells, central memory, effector memory CD4^+^ and CD8^+^ T cells, Tγδ cells, T helper 1 (Th1) cells, Th2 cells, Th17 cells, Tregs, Tfh cells, and activated, immature, and memory B cells. The innate immune system comprises macrophages, monocytes, mast cells, eosinophils, neutrophils, activated plasmacytoid dendritic cells (DCs), immature DCs, NK cells, NKT cells, and myeloid-derived suppressor cells (MDSCs).

Furthermore, we examined the correlation between CDH6 expression and the ESTIMATE score and tumor purity. We used the ESTIMATE algorithm to infer stromal and immune cell fractions from TCGA LGG–GBM dataset. The ESTIMATE score positively correlated with CDH6 expression (R = 0.25; *p* = 0) (Figure 5C). Tumor purity negatively correlated with CDH6 expression (R = 0.24; *p* = 0) (Figure 5D). Therefore, we concluded that CDH6 might be related to stroma production and immune infiltration.

**FIGURE 6:**
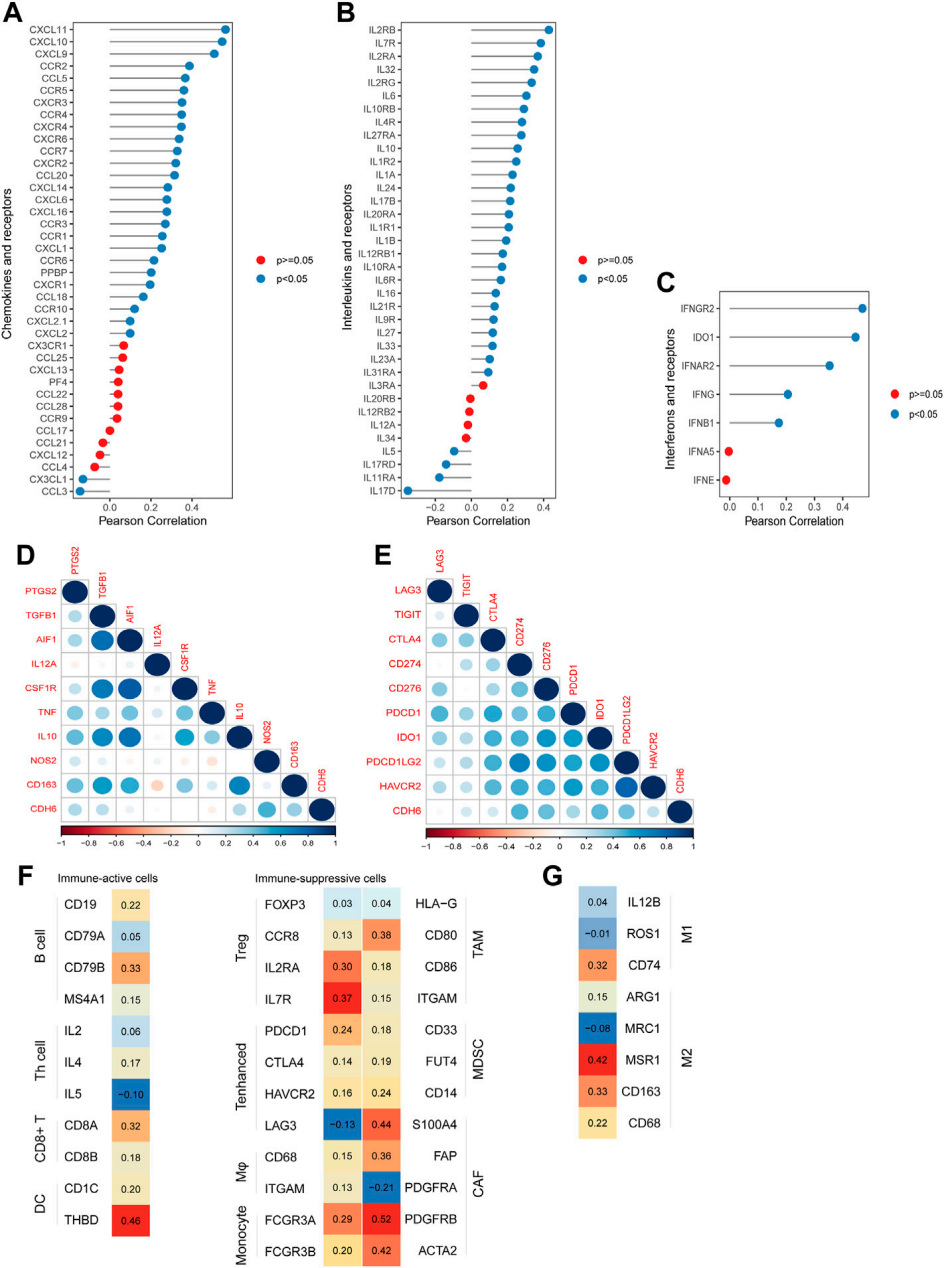
Correlation of CDH6 expression with cytokines and immune cell markers. **(A–C)** Correlation of CDH6 expression with chemokines, interleukins, interferons and their receptors. **(D–E)** Correlation of CDH6 expression with markers of macrophages and immune-related checkpoints. **(F–G)** Association of CDH6 expression with macrophage subtype markers.

Chemokines, interleukins (ILs), interferons (IFNs), and their corresponding receptors play important roles in the induction of inflammatory processes (Figures 6A–C). We examined the correlation between CDH6 and these molecules in TCGA LGG–GBM dataset. CDH6 positively correlated with CCR5, C‐C motif chemokine ligand (CCL) 5, IL12 receptor subunit beta 1, IL2 receptor gamma, IFN gamma receptor 2, and many other cytokines, indicating an important role of CDH6 in signal transmission and different immune processes in the glioma microenvironment.

### Correlations between CDH6 and immune cell markers

To explore the role of CDH6 further in the glioma microenvironment, we evaluated the correlation between CDH6 and the commonly recognized immune cell markers. Nine classical phenotype markers of M0 (allograft inflammatory factor 1), M1 macrophages (IL12 subunit alpha, tumor necrosis factor, nitric oxide synthase 2, and prostaglandin-endoperoxide synthase 2), and M2 macrophages (IL10, CCL163, transforming growth factor-beta 1, and colony-stimulating factor 1R) were analyzed using TCGA LGG–GBM database (Figure 6D). Correlation analysis of CDH6 and nine immune-related checkpoints (PD-1, PD-L1, PD-L2, T cell immunoglobulin and mucin-domain containing 3, lymphocyte-activation gene 3, CTLA4, T cell immunoglobulin and immunoreceptor tyrosine-based inhibitory motif domain, indoleamine 2,3-dioxygenase 1, and CD276) in TCGA LGG–GBM dataset was also performed (Figure 6E). CDH6 strongly correlated with immunosuppressive cells, including Tregs, monocytes, macrophages, tumor-associated macrophages (TAMs), and MDSCs. It also showed correlations with immune-active cells, such as B cells, CD8^+^ T cells, and DCs (Figures 6F,G). Furthermore, CDH6 exhibited both positive and negative correlations with markers of Th cells, enhanced T cells, cancer-associated fibroblasts, and M1 and M2 macrophages. Considering the complexity of the intercellular and molecular mechanisms underlying the glioma microenvironment, it was difficult to conclude the role of CDH6 through the aforementioned analyses. Future closer-in investigations of CDH6 with certain molecules or intercellular mechanisms are needed.

### CDH6 in prediction of drug response

**FIGURE 7:**
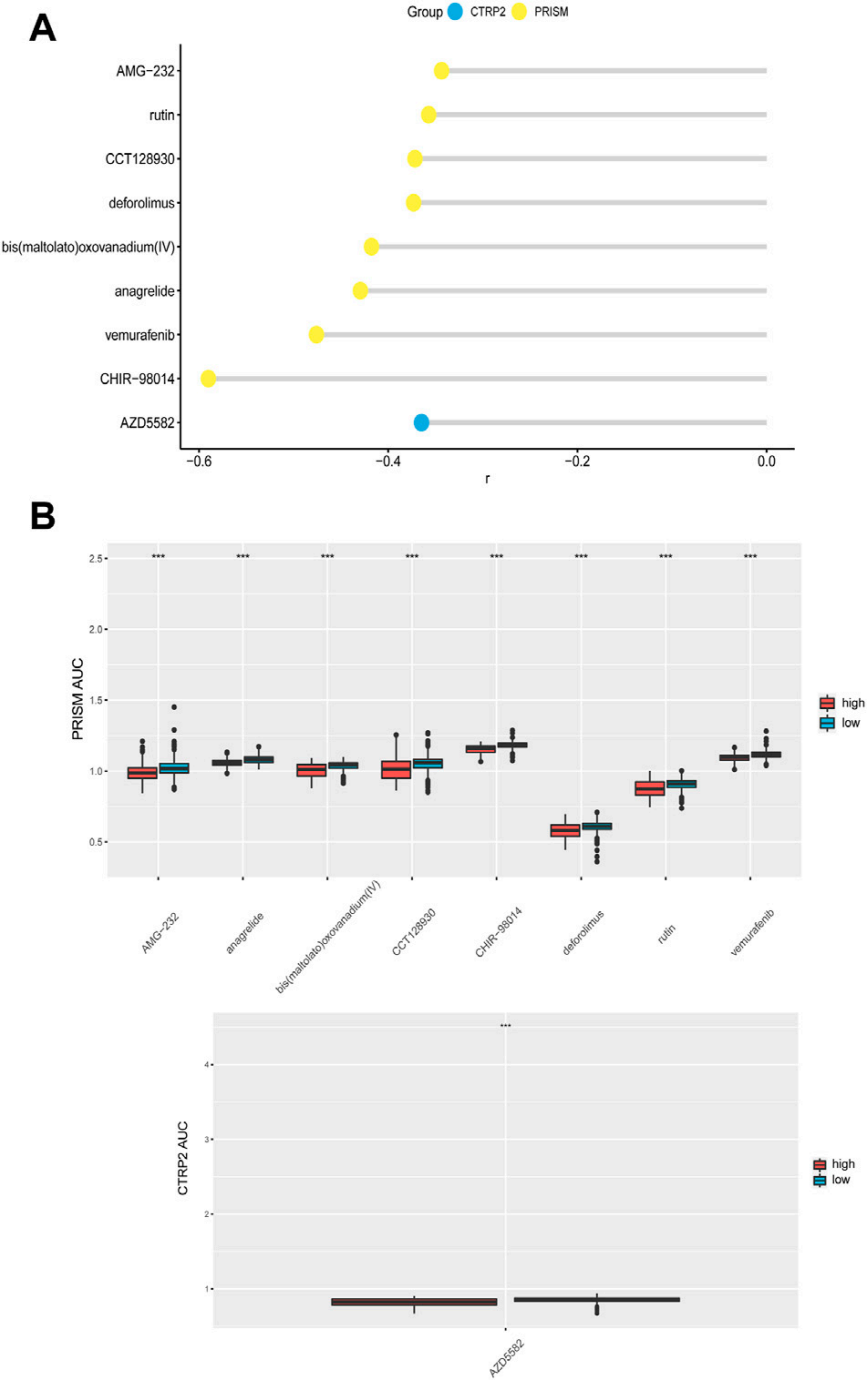
CDH6 in predicting drug response. **(A)** Correlation of CDH6 expression and AUC value of potential drugs. **(B)** Distribution of the AUC values of drugs in CDH6 expression subgroups. *p < 0.05, **p < 0.01, ***p < 0.001.

We evaluated the effectiveness of CDH6 in predicting drug responses using data from the PRISM and Cancer Therapeutics Response Portal (CTRP) databases. Drugs with significantly different area under the curve (AUC) values between the high and low CDH6 expression subgroups and Spearman correlation “r” >0.3 were filtered out. The evaluated drugs included AMG-22, rutin, CCT128930, deforolimus, bis(maltolato)oxovanadium, anagrelide, vemurafenib, CHIR-98014, and AZD5582 (Figures 7A,B).

### CDH6 expression in astrocyte-like malignant cells trajectory

**FIGURE 8:**
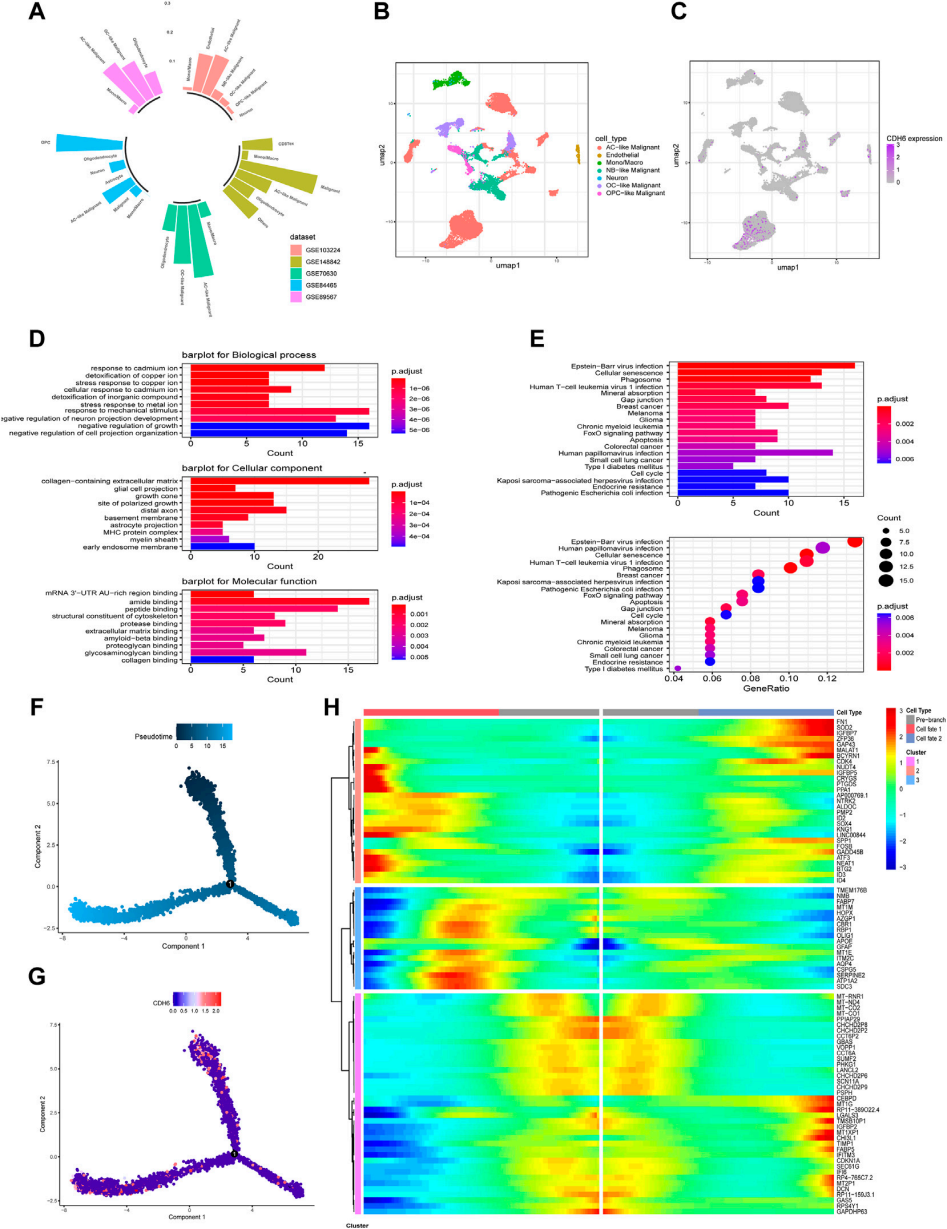
CDH6 in a single-cell point of view. **(A)** Summary of CDH6 expression in five single cell datasets. **(B,C)** Cell clustering and CDH6 expression according to TISCH preprocessing and analysis. **(D,E)** GO and KEGG analyses of differentially expressed genes between AC-like malignant cells and other malignant cells. **(F,G)** Pseudotime analysis of AC-like malignant cells and corresponding CDH6 expression in each cell. **(H)** Different expression patterns of AC-like malignant cell subclusters.

Tumor Immune Single-cell Hub (TISCH) is an online tool that harbors single-cell datasets for different cancer types and provides metadata, cell type annotation, and several other analysis methods. We examined CDH6 expression in five glioma single-cell datasets (Figure 8A) using TISCH. CDH6 was expressed mainly in a cluster of cells annotated as AC-like malignant cells (Figures 8B,C). Differentially expressed genes were identified between AC-like and other malignant cells (|log2FC|>1; adjusted *p*-value<0.01). GO and KEGG analyses identified differentially expressed genes to be enriched in pathways associated with stress response, membranous processes, viral infections, and several cancer types (Figures 8D,E). We then performed trajectory analyses of AC-like malignant cells using the Monocle2 algorithm (Figures 8F,G). Gene changes in different sub-clusters of AC-like malignant cells are shown in Figure 8H.

## Materials and methods

### Data acquisition

TCGA LGG–GBM dataset was obtained from the UCSC Xena website (https://xenabrowser.net/). RNA-sequencing data from 702 cases were included in the dataset. The corresponding clinical information was obtained from the UCSC Xena website. Another dataset was obtained from the CGGA website that included 325 cases of glioma. Clinical specimens were collected from surgical patients diagnosed with glioma at the Xiangya Hospital, Central South University. This study was approved by the ethics committee of Xiangya Hospital (No. 201703478).

### Functional analyses

GO and KEGG pathway enrichment analyses were performed using clusterProfiler R package to evaluate the biological processes associated with CDH6 expression (Benjamini–Hochberg adjusted *p*-value<0.01). GSVA was performed to acquire the individual immune function scores for each case in the datasets.

### Immune infiltration analyses

The CIBERSORTx online tool (https://cibersortx.stanford.edu/) was utilized to calculate the infiltration of the 22 types of immune cells. The correlation between CDH6 expression and immune cell infiltration was evaluated. The estimate R package was used to calculate the stromal, immune, and ESTIMATE scores of each case in the datasets. The stromal score reflects the stromal cell proportion in the tumor bulk. Immune score indicates the level of immune cell infiltration. The ESTIMATE score determines the tumor purity.

### Co-expression module identification

WGCNA was used to identify modules that significantly correlated with CDH6 expression. The top 10,000 genes with the highest MAD values were used in the WGCNA. Soft thresholding power was selected based on the criterion of an approximate scale-free topology. The minimum cut size was set at 30 and cut height at 0.25 for network construction and consensus module detection. Genes in the turquoise, green, and purple modules were selected for the enrichment analyses. Cytoscape software was used for protein–protein interaction network visualization.

### Potential drug prediction

Drug sensitivity and corresponding expression information were obtained from PRISM repurposing dataset (https://depmap.org/portal/prism/) and CTRP (https://portals.broadinstitute.org/ctrp). Drug sensitivity was denoted by low AUC values indicative of high drug sensitivity. The AUC values of the samples in this study were calculated using “pRRophetic” R package.

### Single-cell sequencing analysis

TISCH is a single-cell RNA-sequencing (scRNA-seq) database that aims to characterize the tumor microenvironment at single-cell resolution (Sun et al., 2021). The preprocessed scRNA-seq expression matrix and metadata, including cell annotations, were downloaded from TISCH. Differential analysis between cell clusters was performed using the Wilcoxon rank-sum test with FindMarkers function in Seurat R package (adjusted *p*-value<0.01; logfc. threshold = 1). Monocle R package was used for pseudotime analysis.

### Real-time quantitative polymerase chain reaction

Total RNA was extracted using TRIzol reagent. The reverse transcription reaction was performed using RevertAid First Strand cDNA Synthesis Kit (Thermo Fisher). ChamQ universal SYBR qPCR Master Mix (Vazyme, China) and StepOne Real-time PCR systems (Applied Biosystems) were used for real-time quantitative polymerase chain reaction. Primer sequences were designed in the laboratory and were synthesized using TsingKe Biotech. The expression levels were calculated using the 2^−ΔΔCt^ method. The primer sequences used are listed in Supplementary Table S1.

### Statistical analysis

Statistical analysis was performed using R software (version 4.1.3). Immune cell infiltration was calculated using TIMER algorithm. The ESTIMATE, stromal, and immune scores were computed using the ESTIMATE algorithm. Spearman correlation was utilized to evaluate the correlation between variables. Survival analysis was performed using the Kaplan-Meier method; *p*-values<0.05 were considered statistically significant.

## Discussion

CDH6 is an EMT marker that is highly expressed in solid tumors and that facilitates tumor invasiveness and metastasis. It has been implicated in renal carcinomas and correlates with lymph node invasion and metastasis (Paul et al., 1997). Studies have also found CDH6 expression in cases of ovarian carcinoma (Köbel et al., 2008) and thyroid cancers (Zhao et al., 2016). In osteosarcoma, CDH6 overexpression reportedly correlated with overall survival and patient prognosis. Additionally, CDH6 correlated with stem-cell-related transcription factors, including FOXM1, SNAI1, SOX9, and MCM2, in triple-negative breast cancer.

This study focused on the role of CDH6 in gliomas. The significantly varying levels of expression in gliomas of different pathological grades indicate an underlying role of CDH6 in glioma genesis and progression. The significantly high expression of CDH6 in classical and mesenchymal subtypes implied a correlation between CDH6 and certain biomarkers of these two subtypes. Pathway enrichment analyses indicated the involvement of CDH6 in multiple biological processes, including cell–cell adhesion, axon guidance, extracellular matrix constituents, transmembrane transporter activity, and several cancer types. Our results corroborated those of previous studies, indicating a significant role of CDH6 in breast and thyroid cancers and its correlation with Hippo and Wnt signaling pathways and stem cells. GSVA revealed a correlation between CDH6 and multiple immune-related pathways, cell–cell interactions, angiogenesis, and cell cycle. Therefore, we examined the correlation between CDH6 expression and immune processes.

CDH6 expression correlated with the ESTIMATE score and tumor purity. Individual immune cell types were examined for their respective correlations with CDH6 expression. The infiltration levels of M0, M1 macrophages, monocytes, activated NK cells, Tregs, Tγδ cells, and Tfh cells significantly correlated with CDH6 expression. Immune cells have been implicated in the glioma microenvironment, and they may affect the therapeutic response. The correlations between CDH6 and immune cell markers were also examined. Correlations between CDH6 and markers of B cells, DCs, CD8^+^ T cells, macrophages, monocytes, Tregs, MDSCs, and TAMs were uniformly positive.

Potential drugs were predicted for the high CDH6 expression subgroup. Rutin has been reported to enhance temozolomide efficacy by inhibiting c-Jun N-terminal kinase-mediated autophagy in GBM (Zhang et al., 2017). CCT128930 induces apoptosis and cell cycle arrest in human osteosarcoma cells (Wang et al., 2014). Deforolimus decreases the mammalian target of rapamycin pathway activation and inhibits glioma growth in certain subtypes (Hütt-Cabezas et al., 2013). Anagrelide inhibits GBM cell migration *in vitro* in an L1-dependent manner (Nagaraj et al., 2022). Vemurafenib demonstrated durable antitumor activity in some patients with *BRAF* ^V600^-mutant gliomas (Kaley et al., 2018). The glycogen synthase kinase-3 inhibitor, CHIR-98014, downregulates sonic hedgehog (SHH)-driven proliferation in cerebellar neurogenesis and may be useful in treating SHH-driven medulloblastomas (Ocasio et al., 2019). AZD5582, an anti-apoptotic protein inhibitor, activates glioma cell apoptosis when carried by liposomes (Kuo et al., 2021).

Considering the aforementioned role of CDH6 in cell–cell interactions, the underlying molecular mechanism of the correlations between CDH6 and different immune cells warrants further investigation. CDH6 expression was also examined in single-cell resolution and was mainly expressed in a cluster of cells annotated as AC-like malignant cells. GO and KEGG enrichment analysis of differentially expressed genes between AC-like and other malignant cells identified pathways associated with cellular interactions, morphogenesis, various cancers, and apoptosis. Further pseudo-time studies identified the subtypes in AC-like malignant cells, and cells with high CDH6 expression were concentrated in two of the three subtypes. Additionally, genes related to AC-like malignant cell subdivision were identified. Single-cell studies on the gene regulatory network and cell–cell communication are needed to clarify the role of CDH6 further in glioma malignancy and the microenvironment.

## Data Availability

The datasets presented in this study can be found in online repositories. The names of the repository/repositories and accession number(s) can be found in the article/Supplementary Material.
