# Supplementary material for: CDH6 as a prognostic indicator and marker for chemotherapy in gliomas
Source: Front Genet. 2022 Jul 22;13:949552. doi: 10.3389/fgene.2022.949552 (PMC9355303; doi:10.3389/fgene.2022.949552)
Supplement: Supplementary file 1 [file Table1.DOCX]

| **Gene Symbol** | **Forward primer(5->3)** | **Reverse primer(5->3)** |
| --- | --- | --- |
| GAPDH | CCTCACAGTTGCCATGTAGA | TGGTACATGACAAGGTGCG |
| CDH2 | GTACAGAATCAGTGGCGGA | TTGGTTTGACCACGGTGACTAA |
| CDH9 | ATGTTCCATACAGTTGACAC | CTTTTGTCAGACCCGCTA |
| CDH7 | TATTCTGCAAGGACAGCCGTA | ACTGGTCTTTAGCCTCTCTATC |
| CDH6 | CCAGTGGCTCAAACTTTACC | AATGACCACAGGCAAGAGATA |
| CDH5 | CGATAACACGGCCAACAT | CTTGGCATCCCATTGTCTGA |
| CDH3 | CTGTGTCAGAGAATGGTGC | GGGTAAACTTGGGCTTGT |
| CDH10 | ACCGAATTATTGATGGTGACG | CTCTCATAGTCGAGTGGCT |
| CDH11 | CAATGTGGGAACGTCAGTAAT | GTACACTAACTTGGCGCTAT |
| CDH12 | GAAGAAATGGATACAGCCGCA | AGAGTCACATCTACAGACTCG |
| CDH13 | TCAGCAGAAAGTGTTCCATATC | AGTCACTGAAGGTCAAGTTTAG |
| CDH18 | AACATCACAGTCACTGCTTC | TCATATTCCCTGGCAAGTTCG |
| CDH20 | TGGACTTCTGGTAGAATGAGC | GGTGGTCGTAAGGTCCATC |
| CDH22 | GGAACCAGTTCTTCGTGGTA | CTCGTCTGAGTCGGAGTG |
| CDH23 | TCACCATCGAGGTGTTTGA | GCTCCTGCCATGATGTTC |
| CDH26 | TCCGGTGGAAGGAAGGAT | GCTGTAGACGTGAGGTAGG |
