# Supplementary figures and images for: CDH6 as a prognostic indicator and marker for chemotherapy in gliomas
Source: Front Genet. 2022 Jul 22;13:949552. doi: 10.3389/fgene.2022.949552 (PMC9355303; doi:10.3389/fgene.2022.949552)

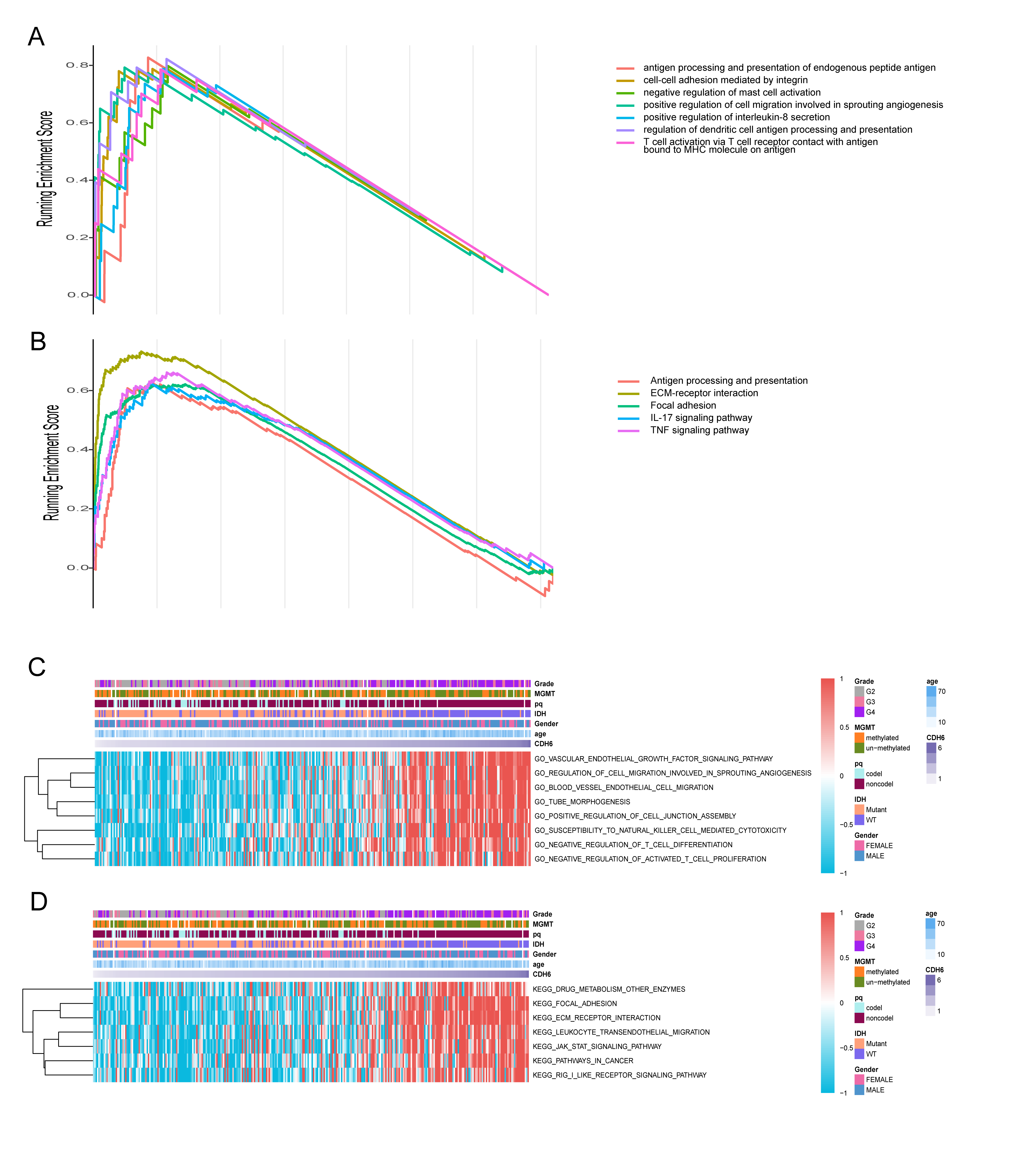

Supplement: Supplementary file 2 [file Image3.TIF]

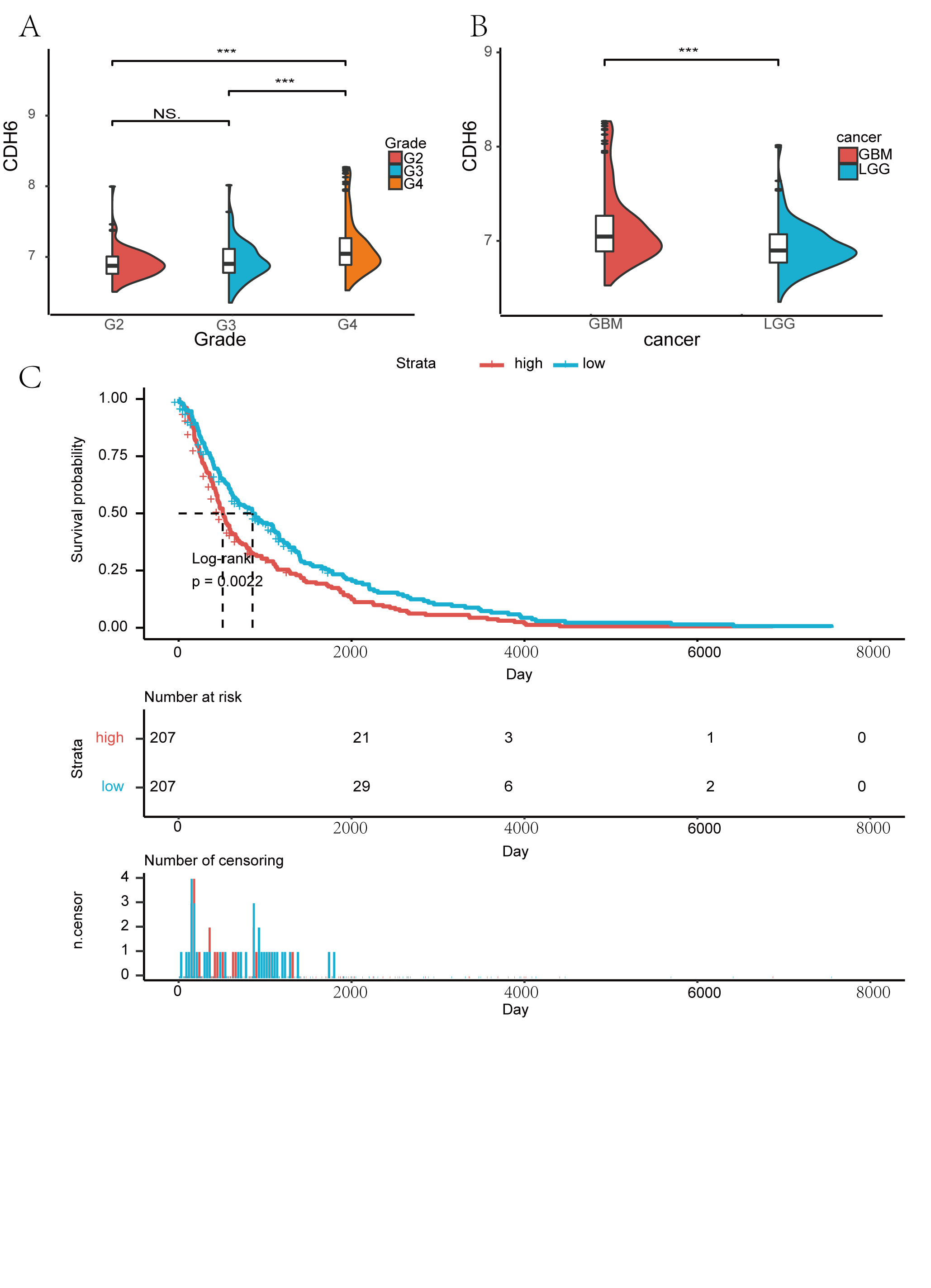

Supplement: Supplementary file 3 [file Image2.TIF]

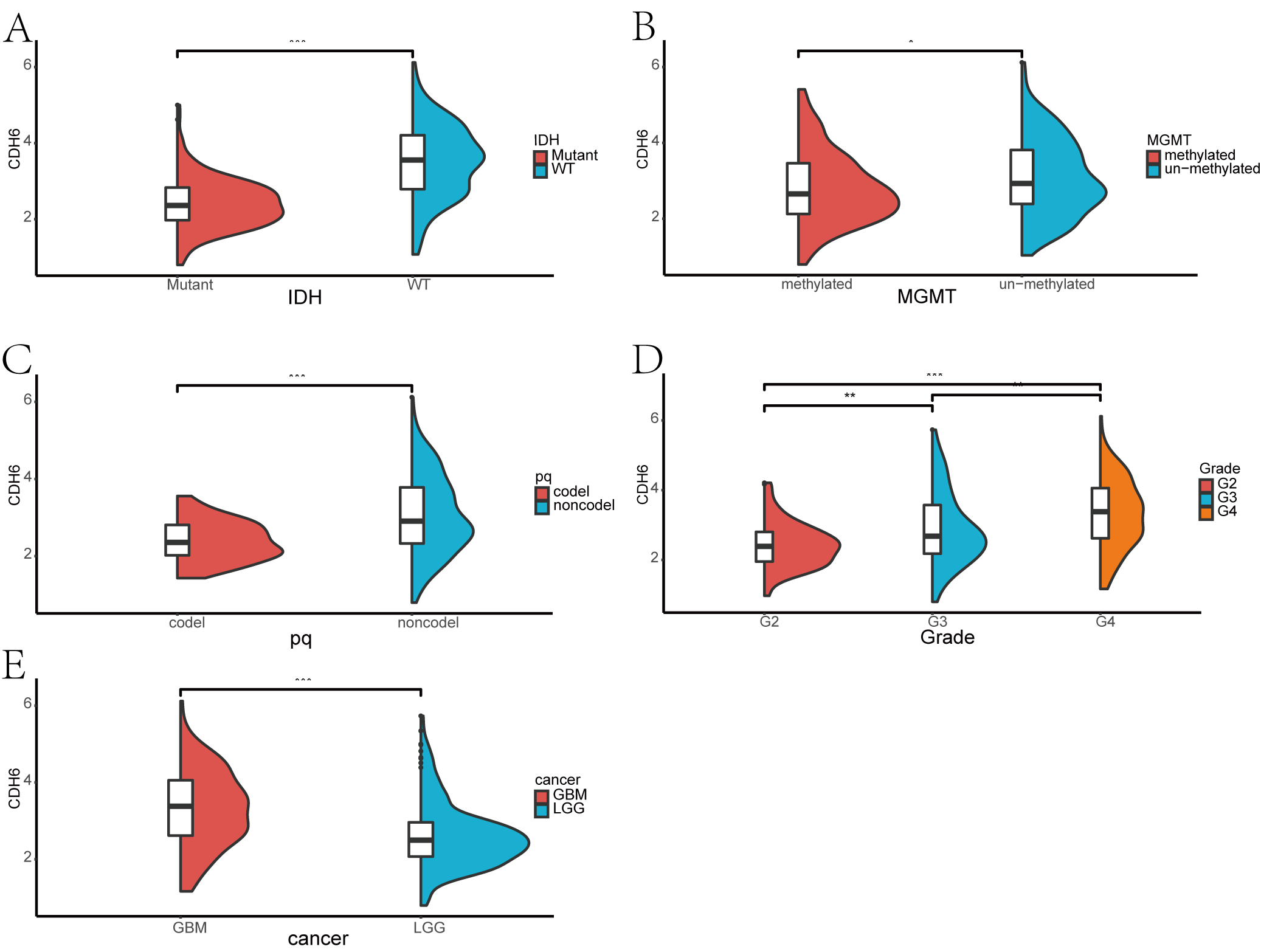

Supplement: Supplementary file 4 [file Image1.TIF]
